# The Role of the Triglyceride–Glucose Index and Other Prognostic Factors in Predicting Coronary Slow Flow

**DOI:** 10.3390/jcdd13010055

**Published:** 2026-01-20

**Authors:** Fethullah Kayan, Halil Kömek, Ferat Kepenek, Mehmet Serdar Yildirim

**Affiliations:** 1 Department of Cardiology, SaglikBilimleri University Diyarbakir Gazi Yasargil Training and Research Hospital, 21070 Diyarbakir, Turkey; 2 Department of Nuclear Medicine, SaglikBilimleri University Diyarbakir Gazi Yasargil Training and Research Hospital, 21070 Diyarbakir, Turkey; halilkomek@gmail.com (H.K.); feratkepenek@hotmail.com (F.K.); 3 Department of Internal Medicine, SaglikBilimleri University Diyarbakir Gazi Yasargil Training and Research Hospital, 21070 Diyarbakir, Turkey

**Keywords:** coronary slow flow, triglyceride–glucose index, insulin resistance, TIMI frame count, coronary angiography

## Abstract

Background: Insulin resistance (IR) has been implicated in cardiovascular diseases, and a correlation between IR and the slow flow phenomenon (CSF)has been identified. The triglyceride–glucose index (TGI), a simple surrogate marker for IR, has recently emerged as a potential predictor of CSF, though data are limited. The aim of this study was to evaluate the association of TGI and other prognostic parameters in patients with CSF. Methods: This retrospective study included 693 patients who underwent diagnostic coronary angiography between January 2022 and December 2024. A total of 132 patients were diagnosed with CSF based on the corrected TIMI frame count (cTFC > 27 in at least one epicardial coronary artery), while 561 patients had normal coronary flow (NCF). Patients with confounding cardiovascular or systemic conditions were excluded. Clinical, demographic, and laboratory data were gathered, and TGI was calculated as ln [fasting triglycerides (mg/dL) × fasting glucose (mg/dL)/2].Results: Statistically significant distinctions were found between the CSF and NCF groups regarding TGI, age, glucose, HbA1c, creatinine, sodium, CRP, platelet count, heart rate, PR interval, and cQT interval (*p* < 0.05). Age, hypertension, diabetes mellitus, HbA1c, glucose, sodium, and cQT were identified as potential clinical and laboratory factors associated with CSF in univariate logistic regression analysis; however, no independent predictor was found in multivariate analysis. ROC analysis showed that a TGI cut-off value of ≥8.93 predicted CSF with 67.6% sensitivity and 66.7% specificity. Conclusions: Our study demonstrated that TGI was significantly greater in patients with CSF compared to those with NCF. Although TGI showed limited sensitivity and specificity in discriminating CSF, its possible value as a prognostic indicator warrants further validation in prospective, large-scale studies.

## 1. Introduction

Coronary slow flow (CSF) was first described by Tambe et al. in 1972 and is characterized by delayed opacification of the epicardial coronary arteries in the absence of significant coronary stenosis [1,2]. The Thrombolysis in Myocardial Infarction Frame Count (TFC) method is used for the quantitative assessment of CSF [3]. The prevalence of CSF in patients undergoing diagnostic coronary angiography due to suspected coronary artery disease ranges from 1% to 7% [4]. CSF is a relatively common phenomenon and has been associated with adverse cardiovascular events and life-threatening arrhythmias [5]. Although its clinical presentation is complex and variable, the most typical symptom is recurrent resting angina attacks, which significantly impair quality of life [6]. Although the exact pathophysiology of CSF remains unclear, endothelial and microvascular dysfunction, in conjunction with inflammation, are believed to play a role in its development [7]. Specifically, atherosclerosis results from the progressive accumulation of low-density lipoprotein cholesterol (LDL-C) and other apolipoprotein-B (ApoB)-containing lipoproteins in the arterial wall, triggering a series of inflammatory responses that determine the formation and progression of atherosclerotic plaque. Acute and/or chronic inflammation also exacerbates endothelial dysfunction and accelerates atherogenesis by affecting the epicardial and microvascular coronary bed. Myocardial ischemia and hypoxia due to atherogenesis sometimes occur in the microvascular bed, even though the epicardial coronary arteries remain open in the coronary bed. The European Society of Cardiology (ESC) guidelines recommend anti-inflammatory, lipid-lowering, antidiabetic, and anti-obesity drugs for hypoperfusion and ischemia, including structural and/or functional changes affecting the epicardial or microvascular region. This medical treatment is crucial for optimizing clinical outcomes and preventing cardiovascular events. Furthermore, therapeutic strategies targeting metabolic and inflammatory pathways (e.g., lifestyle modifications, statins, GLP-1 receptor agonists) are gaining importance in managing microvascular dysfunction [8].Insulin resistance (IR) has been confirmed as a key contributor to the pathophysiology of cardiovascular diseases (CVD) [9], and a correlation between IR and CSF has also been reported [10].

The triglyceride–glucose index (TGI), calculated using fasting triglyceride and glucose levels, has been reported to be significantly associated with insulin resistance and has been proposed as a reliable surrogate marker for IR [11]. Recently, promising results have been published regarding the use of TGI in discriminating CSF. However, these studies remain limited in number and scope [12,13]. The aim of this study is to investigate the role of TGI and other clinical and laboratory factors in predicting CSF.

## 2. Materials and Methods

### 2.1. Study Design and Patient Selection

Our retrospectively designed study included patients who underwent coronary angiography, for any reason, between January 2022 and December 2024 and were over 18 years of age. Patients with slow coronary flow and patients with normal coronary flow were included in the study. Patients were excluded from the study if they had a history of myocardial infarction (MI), coronary artery bypass grafting (CABG), or percutaneous coronary intervention (PCI); coronary stenosis > 40%; heart failure; advanced valvular heart disease; incomplete or inaccessible data; malignancy; use of statins or triglyceride-lowering agents; severe left ventricular hypertrophy(LVH); severe hepatic disease; renal failure; myocardial bridging; or atrial fibrillation(AF).

Thisstudy was conducted in accordance with current legal regulations and Good Clinical Practice (GCP) guidelines, following approval by the local ethics committee (approval number: 451; dated 9 May 2025).

### 2.2. Data Collection

Patient records were reviewed through the hospital information system. Demographic and clinical characteristics, including age, sex, medical history, and laboratory findings, were recorded. Blood samples were collected after a 12 h fasting period, and biochemical results were documented accordingly.

### 2.3. Calculation of the Triglyceride–Glucose Index (TGI)

The TGI was calculated using the following formula [11,14]:

TGI = ln [fasting triglycerides (mg/dL) × fasting glucose (mg/dL)/2]

### 2.4. Coronary Angiography

Coronary angiography was performed by an interventional cardiologist using the Judkins technique via femoral or radial access with a PHILIPS angiography system. The contrast agents used were either iopromide (Ultravist-370) or iohexol (Omnipaque).

Coronary arteries were visualized in cranial and caudal projections of right and left oblique views, recorded at 30 frames per second (fps). The starting point was defined as the moment the contrast first fully opacified the coronary ostium, while the endpoint varied according to the artery: for the right coronary artery (RCA), it was the appearance of the first lateral branch of the posterolateral artery; for the left anterior descending artery (LAD), it was the so-called “mustache” landmark; and for the left circumflex artery (LCx), it was when the contrast reached the distal bifurcation of the longest branch. For LAD and LCx, right anterior oblique (RAO) or left anterior oblique–caudal (LAO–caudal) views were used as references; for RCA, a left anterior oblique–cranial view was used. TIMI frame count (TFC) was calculated by analyzing digitally stored angiographic images in DICOM format. The corrected TIMI frame count (cTFC) for the LAD was obtained by dividing the raw frame count by 1.7. The mean TFC for each patient was calculated by averaging the corrected frame counts from the LAD, LCx, and RCA [15]. Frame counting was performed by two experienced interventional cardiologists who were blinded to the patients’ clinical and laboratory data. Inter-observer variability was assessed and showed good agreement (Intraclass Correlation Coefficient >0.90). The use of mean cTFC was adopted to provide a single, integrated measure of overall coronary flow velocity for each patient, as commonly reported in the CSF literature.

According to the current definition proposed by Beltrame, the coronary slow flow phenomenon (CSFP) is defined as a TIMI grade 2 flow or a corrected TIMI frame count (cTFC) exceeding 27 frames in one or more epicardial coronary arteries [15].

In this study, patients were classified as having CSFP based on this definition.

### 2.5. Statistical Methods

Statistical analysis was made using IBM SPSS Statistics for Windows, version 25.0 (IBM Corp., Armonk, NY, USA). Continuous variables were tested for normality using the Kolmogorov–Smirnov test. Data are presented as means ± standard deviations (SDs) for normally distributed variables, and as medians (interquartile ranges [IQRs] or min-max) for non-normally distributed variables. Categorical variables are presented as frequencies and percentages. Group comparisons were performed using Student’s t-test or Mann–Whitney U test for continuous variablesand the chi-square or Fisher’s exact test for categorical variables. Standardized mean differences (SMD) were calculated for key variables to report effect sizes. Due to the exploratory nature of the study and the large number of comparisons, findings should be interpreted with caution. Univariate logistic regression analysis was performed to identify variables associated with CSF. Variables with a *p*-value < 0.10 in univariate analysis and those deemed clinically relevant were entered into a multivariate logistic regression model using the enter method to identify independent predictors. Model performance was assessed using the area under the receiver operating characteristic curve (AUC), the Hosmer–Lemeshow goodness-of-fit test, and Nagelkerke R^2^. Multicollinearity was checked using variance inflation factors (VIFs). For variables with significant missing data (e.g., TGI, HbA1c), a complete-case analysis was employed for the regression models. The characteristics of patients with and without missing TGI data were compared to assess potential bias. Receiver operating characteristic (ROC) curve analysis was used to evaluate the discriminative ability of TGI and HbA1c for CSFand to determine optimal cut-off values based on the Youden index. Sensitivity, specificity, and AUC with 95% confidence intervals (CIs) were reported. Internal validation using bootstrapping was performed to assess the stability of the ROC-derived cut-off values. A *p*-value less than 0.05 was considered statistically significant for the final multivariate model and primary comparisons.

## 3. Results

Of the 693 patients included in the study, 391 (56.4%) were female, and the mean age was 57.2 years (range: 23–87). The demographic, clinical, and laboratory characteristics of the patients are presented in Table 1.

Statistical analysis using the Mann–Whitney U test demonstrated that TGI values were significantly higher in the CSF group compared with the NCF group (*p* = 0.014). For the remaining variables, appropriate statistical analyses were applied according to data distribution and variable type, revealing that glucose (*p* = 0.03), HbA1c (*p* = 0.012), age (*p* < 0.001), creatinine (*p* = 0.021), sodium (*p* < 0.001), ALT (*p* = 0.036), CRP (*p* = 0.01), platelet count (*p* < 0.001), heart rate (*p* = 0.041), PR interval (*p* = 0.041), and corrected QT interval (QTc) (*p* < 0.001) were all significantly higher in the CSF group than in the NCF group. Furthermore, categorical variables were compared using chi-square or Fisher’s exact tests, showing that the prevalence of hypertension (*p* < 0.001) and diabetes mellitus (*p* < 0.001) was also significantly higher in the CSF group (Table 1).

In univariate logistic regression analysis, age (*p* < 0.001), hypertension (*p* < 0.001), diabetes mellitus (*p* < 0.001), HbA1c (*p* = 0.019), glucose (*p* = 0.005), sodium (*p* < 0.001), and cQT (*p* < 0.001) were identified as potential clinical and laboratory factors associated with CSF. The multivariate logistic regression model included age, hypertension, diabetes, HbA1c, glucose, sodium, QTc, and TGI. The model’s overall performance was modest (AUC = 0.72, 95% CI: 0.67–0.77; Hosmer–Lemeshow test *p* = 0.45). However, none of the variables retained independent statistical significance (all *p* > 0.05).

The results of the univariate and multivariate logistic regression analyses are presented in Table 2 and Table 3, respectively.

**Figure 1:**
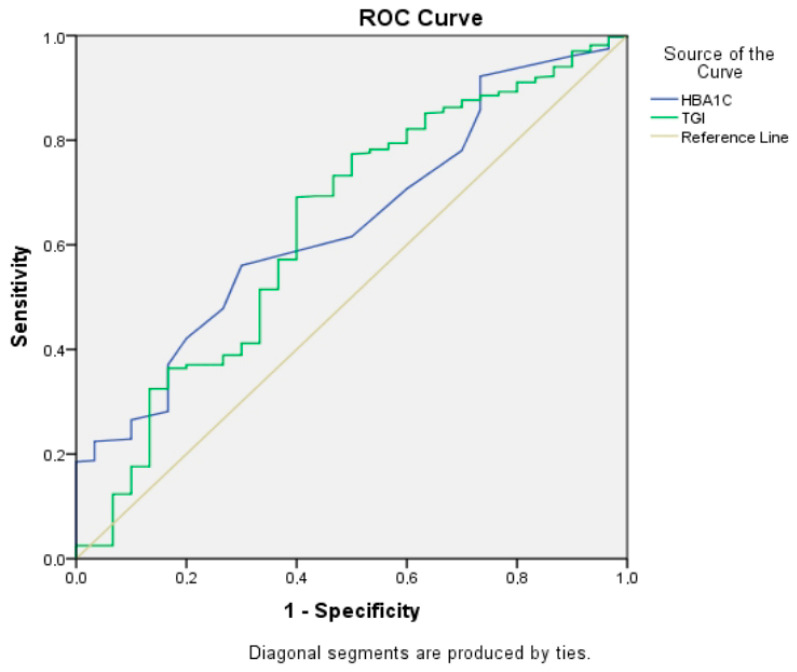
Receiver operating characteristic (ROC) curves illustrating the predictive ability of the TGI variable for coronary slow flow.

The ROC curve analysis revealed that the AUC for TGI in predicting CSF was 0.60 (95% CI: 0.52–0.68). The ROC curve analysis revealed that the cut-off value of TGI for predicting CSF was ≥8.93, with a sensitivity of 67.6% and specificity of 66.7%. Similarly, the cut-off value of HbA1c was found to be ≥6.4 ng/mL, with a sensitivity of 71.8% and specificity of 60% in discriminating CSF (Figure 1). Given the modest AUC and sensitivity/specificity, this cut-off should be considered exploratory and should not be used alone for clinical decision-making.

## 4. Discussion

In this retrospective study, we found that the TGI may serve as associated marker for CSF, and our results demonstrated that TGI was statistically significantly higher in the CSF group compared to the control group. Insulin resistance(IR) has been proposed as a key contributor to cardiovascular disease(CVD) [16].

TGI has recently become a widely used parameter in clinical laboratory research, particularly in studies related to IR-related disorders and CVD [17].

Initially, CSF was described as an angiographic finding of uncertain clinical significance. However, with advances in interventional cardiology, the clinical relevance of CSF is increasingly being recognized [18,19]. Nevertheless, the relationship between TGI and CSF has not been sufficiently investigated [20].

In a retrospective study by Kaplangoray et al., TGI values were found to be significantly higher in the CSF group than in the NCF group (*p* < 0.001) [21]. Similar results have been reported in other studies as well [13,22]. In our study, the Mann–Whitney U test revealed a significant correlation between TGI and CSF (*p* = 0.014). However, no significant association was found in univariate or multivariate logistic regression analyses. The lack of independent predictors in the multivariate model may reflect shared pathophysiological pathways rather than the absence of an association.

In our study, significant clinical and demographic differences were observed between the patient groups with CSF and NCF. According to univariate logistic regression analyses, the CSF group had a significantly higher mean age (*p* < 0.001), and the prevalence of hypertension (*p* < 0.001) and diabetes mellitus (*p* < 0.001) was also significantly greater. However, in the study conducted by Kaplangoray et al [22]., no statistically significant differences were found between the groups in terms of age (*p* = 0.343), hypertension (*p* = 0.37) and diabetes mellitus (*p* = 0.209). Notably, in both our study and that of Kaplangoray et al., serum glucose levels were significantly higher in the CSF group (*p* < 0.001 and *p* = 0.005, respectively). Our study identified several parameters that differed between groups (e.g., age, HT, DM, cQT), which may reflect distinct clinical characteristics of the CSF population rather than causative predictors.

Interestingly, studies evaluating the relationship between the TGI and CSFhave not included corrected QT (cQT) interval and PR interval measurements. However, in our study, the cQT interval (*p* < 0.001) and PR (*p* = 0.041) interval were found to be significantly higher in the CSF group. The association observed between prolonged cQT-PR intervals and CSF is hypothesis-generating and may indicate a potential relationship between CSF and electrical instability–abnormal conduction, warranting further investigation in dedicated studies. On the other hand, some statistically significant differences (e.g., ALT (18 vs. 19), sodium (139 vs. 140), or heart rate (71.5 vs. 76)) may not be clinically significant and should be approached with caution.

In contrast to our study, in a similar study conducted by Yasin Yuksel et al. in 2023, HDL-C, hemoglobin, and TGI were found to be independent factors associated with CSF in multivariate logistic regression analysis [22].These different results need to be clarified by conducting more studies at more centers.

As explained in detail in the Introduction Section, CSF and INOCA (Ischemia with Non-Obstructive Coronary Arteries) are two very similar conditions. Although the pathophysiological causes, clinical presentations, and treatment approaches of these two conditions are similar, CSF is sometimes the cause of INOCA. In a study conducted by Wen Zhang et al. in patients with INOCA in 2024,the incidence of major adverse cardiac events (MACEs) was significantly increased in the group with high TGI compared to low TGI. TGI emerged as an independent prognostic factor related to unfavorable prognosis in INOCA patients. The prognostic importance of TGI in predicting both myocardial ischemia and MACE formation in individuals with INOCA was emphasized [23]. The results of this study seem to support the finding that TGI can be used as an associated marker of CSF in our study.

In addition, independent of CSF, TGI is generally accepted as a marker of cardiovascular disease. In a study conducted by Susilane Pereira Araújo and colleagues in 2022, TGI hada good predictive capacity in predicting ten-year cardiovascular risk assessed according to the Framingham risk score. In this context, a TGI value of 9.04, with a sensitivity of 62.5% and a specificity of 66.7%, was determined as the cut-off point [24].

In 2020, Gyung-Min Park et al. used the TGI to detect coronary artery disease(CAD) in patients without cardiovascular risk factors (defined as ±systolic/diastolic blood pressure ≥ 140/90 mmHg; fasting glucose ≥ 126 mg/dL; total cholesterol ≥ 240 mg/dL; low-density lipoprotein cholesterol ≥ 160 mg/dL; high-density lipoprotein cholesterol < 40 mg/dL; body mass index ≥ 25.0 kg/m^2^; current smoking; and previous medical history of hypertension, diabetes, or dyslipidemia). Patients were divided into three groups according to the TGI value. The prevalence of CAD increased with increasing TGI tertiles (group I: 14.8% vs. group II: 19.3% vs. group III: 27.6%; *p* < 0.001). Multivariate logistic regression models showed that TGI was associated with an increased risk of CAD. The most appropriate TGI threshold for predicting CAD was 8.44 (sensitivity 47.9%; specificity 68.5%) [25].

In parallel with the studies conducted by Susilane Pereira Araújo et al. and Gyung-Min Park et al. [24,25], our study identified a TGI cut-off value of ≥8.93, with a sensitivity of 67.6% and a specificity of 66.7%, for the detection of CSF.

In a study by Bilen et al., in patients with CSF, the cut-off value for TGI for predicting CSF was determined as ≥4.76, with a sensitivity of 88% and specificity of 89% [12]. In contrast, our study identified the TGI cut-off value as ≥8.805, with a sensitivity of 67.6% and specificity of 66.7%.

In conclusion, in the context of cardiovascular diseases in general—and more specifically in subgroups such as CSF, INOCA, and cases with intermediate-to-high Framingham risk scores—studies evaluating the sensitivity, specificity, and cut-off values of the TGI have yielded varying results. To establish a more definitive and reliable cut-off value for the TGI, along with higher sensitivity and specificity, large-scale, multicenter studies involving a greater number of participants are needed.

### Limitations of Our Study

The most significant limitations of our study are its retrospective design and the relatively small sample size. Additionally, the inability to generalize the findings to a broader population represents another important limitation. In addition, insulin levels and HOMA-IR were not available, preventing direct quantification of IR; the retrospective single-center design and extensive exclusion criteria may introduce selection bias and limit external validity; missing data for several variables may contribute to information bias; and multiple univariate comparisons increase the risk of type I error. Residual confounding cannot be excluded.

## 5. Conclusions

In this retrospective study, we observed that the TGI is associated with CSF; however, it did not show independent association in multivariable analysis, and its discriminatory performance was modest. Prospective, multicenter studies are required before clinical use for risk stratification.

## Figures and Tables

**Table 1:** Baseline demographic, clinical, and laboratory characteristics of the study population.

	NCF		CSF		TOTAL		
	*n*	Median(Min–Max)	*n*	Median(Min–Max)	*n*	Median	*p*
Age	561	51.5 (23–87)	132	59 (23–82)	693	57 (23–87)	<0.001
TGI	441	7.93 (7.49–11.43)	130	8.93 (7.63–10.83)	471	8.11 (7.49–11.43)	0.014
EF	521	60 (45–65)	128	60 (45–65)	649	60 (45–65)	0.06
LAD	423	36 (20–41)	126	37 (34–47)	549	36 (20–47)	0.98
Heart rate	411	71.5 (48–115)	128	76 (50–106)	539	76 (48–115)	<0.001
Glucose	559	98.5 (51–113)	132	103 (68–375)	691	101 (51–375)	0.03
Creatinine	559	0.71 (0.42–10.1)	132	0.76 (0.39–7.3)	691	0.75 (0.39–10.1)	0.021
Na	559	139 (126–150)	132	140 (134–151)	691	139 (126–151)	<0.001
K	559	4.33 (3.19–4.7)	132	4.31 (3.63–5.9)	691	4.32 (3.19–5.9)	0.67
AST	559	20 (7–185)	132	20 (9–85)	691	20 (7–185)	0.966
ALT	559	18 (6–213)	132	19 (5–334)	691	18 (5–334)	0.036
ALBUMİN	524	41.95 (4.3–51)	108	42 (4.4–55)	632	42 (4.3–55)	0.261
T-Cholesterol	542	181 (77–342)	132	175 (92–326)	674	181 (77–342)	0.536
LDL-C	536	105 (19–203)	132	98.5 (33–190)	668	104 (19–203)	0.250
HDL-C	541	42.3 (0–92.2)	132	44 (21.3–147)	673	42.6 (0–147)	0.200
Triglyceride	540	144.5 (22–1367)	132	154.5 (29–670)	672	146 (22–1367)	0.857
HbA1c	448	5.65 (4.3–13.1)	130	6.4 (4.5–7.1)	578	5.8 (4.3–13.1)	0.012
CRP	545	2 (0.1–146)	109	3.8 (0.3–80)	654	2 (0.1–146)	<0.001
WBC	558	8 (3.31–81.7)	132	8.24 (4.82–15.77)	690	8.04 (3.31–81.7)	0.183
HGB	559	14.1 (8.1–18.3)	132	13.95 (8.4–17.3)	691	14.1 (8.1–18.3)	0.262
HCT	559	43.3 (25–57.7)	132	42.5 (27.2–53.2)	691	43 (25–57.7)	0.320
PLT	559	256 (88–455)	132	291.5 (134–579)	691	260 (88–579)	<0.001
MCV	461	87.5 (59–110.4)	130	86.2 (62.8–96.6)	591	87.4 (59–110.4)	0.258
MCHC	462	32.7 (27–89)	130	32.25 (30.1–34.7)	592	32.7 (27–89)	0.062
MPV	461	10.4 (7.6–14)	130	9.95 (8.9–12.4)	591	10.4 (7.6–14)	0.059
Neutrophil	559	4.79 (1.77–31.4)	132	5.09 (2.53–12.74)	691	4.82 (1.77–31.4)	0.320
Lymphocyte	559	2.28 (0.51–40.8)	132	2.38 (0.58–8.75)	691	2.29 (0.51–40.8)	0.194
Monocyte	559	0.48 (0–31.6)	132	0.48 (0.1–1)	691	0.48 (0–31.6)	0.592
RDW-SD	463	44.4 (0–83.4)	130	44.6 (39–54.3)	593	44.5 (0–83.4)	0.406
RDW-CV	461	13.6 (11.8–84.7)	130	13.8 (12.8–17.6)	591	13.6 (11.8–84.7)	0.120
PLC-R	457	29.5 (12.3–52.8)	130	27.1 (18.9–42.6)	587	29.3 (12.3–52.8)	0.079
PR interval	398	145.5 (93–221)	126	155 (119–200)	524	154 (93–221)	0.041
QT	410	383 (328–590)	128	392.5 (322–477)	538	384.5 (322–590)	0.172
cQT	508	410.5 (295–561)	130	445 (365–536)	638	421 (295–561)	<0.001
CRPtoAlb	442	0.05 (0.04–4.29)	130	0.051 (0.044–1.3)	572	0.05 (0.04–4.29)	0.693
NLR	461	2.11 (0.092–23.03)	130	2.1 (1.062–13.79)	591	2.11 (0.09–23.03	0.986
PLR	461	116.24 (80.03–323)	130	107.9 (78.5–356.8)	591	115.7 (80.03–356.8)	0.791
		*n* (%)		*n* (%)		*n* (%)	
HT	Yes	47 (8.3%)		60 (45.4%)		107 (14.3%)	<0.001
	No	514 (91.7%)		72 (54.6%)		586 (85.7%)	
DM	Yes	60 (10.6%)		37 (28%)		97 (13.9%)	<0.001
	No	501 (89.4%)		95 (72%)		596 (86.1%)	
COPD	Yes	54 (11.7%)		4 (13.3%)		58 (87.8%)	0.48
	No	409 (88.3%)		26 (86.7%)		435 (88.2%)	
CKD	Yes	8 (1.7%)		0 (0%)		8 (1.34%)	0.60
	No	455 (98.3%)		130 (100%)		585 (98.4%)	
AF	Yes	19 (4.2%)		5 (3.8%)		24 (4.1%)	0.14
	No	431 (95.8%)		125 (96.2%)		556 (95.9%)	
Dyslipidemia	Yes	6 (1.3%)		1 (0.7%)		7 (1.1%)	0.35
	No	457 (98.7%)		129 (99.2%)		586 (98.9%)	
LVH	Yes	20 (6.6%)		6 (4.76%)		26 (6.06%)	0,20
	No	283 (93.4%)		120 (95.24%)		403 (93.94%)	
LVDD	Yes	260 (61.4%)		83 (65.8%)		343 (62.4%)	0.16
	No	163 (38.6%)		43 (34.2%)		206 (37.5%)	

NCF: normal coronary flow. CSF: coronary slow flow. TGI: triglyceride–glucose index. EF: ejection fraction. LAD: left atrium diameter. Na: natrium(sodium). K: potassium. AST: aspartate aminotransferase. ALT: alanine aminotransferase. T-Cholesterol: total cholesterol. LDL-C: LDL cholesterol. HDL-C: HDL cholesterol. HbA1c: hemoglobin A1C. CRP: C-reactive protein. WBC: white blood cell. HGB: hemoglobin. HCT: hematocrit. PLT: platelet. MCV: mean corpuscular volume. MCHC: mean corpuscular hemoglobin concentration. MPV: mean platelet volume. RDW-SD: red cell distribution width—standard deviation. RDW-CV: red cell distribution width—coefficient of variation. P-LCR: platelet larger cell ratio. PR İnternal: the time from the beginning of the P wave (atrial depolarization) to the beginning of the QRS complex. QT: the time between the start of the Q wave and the end of the T wave. cQT: corrected QT. CRPtoAlb: C-reactive protein/albumin ratio. NLR: neutrophil lymphocyte ratio. PLR: platelet lymphocyte ratio. HT: hypertension. DM: diabetes mellitus. COPD: chronic obstructive pulmonary disease. CKD: chronic kidney disease. AF: atrial fibrillation. LVH: left ventricular hypertrophy. LVDD: left ventricular diastolic dysfunction.

**Table 2:** Univariate logistic regression analyses of factors associated with coronary slow flow.

	B	S.E.	*p*	OR	95% C.I. for EXP(B)	
					Lower	Upper
Age	−0.062	0.009	0.00	0.939	0.921	0.956
Heart rate	−0.015	0.016	0.372	0.985	0.954	1.018
AF	0.962	0.653	0.141	2.617	0.727	9.417
HT	−2.259	0.326	0.00	0.104	0.055	0.198
DM	−1.366	0.317	0.00	0.255	0.137	0.475
Dyslipidemia	0.966	1.097	0.379	2.626	0.306	22.548
CKD	0.809	1.086	0.456	2.246	0.267	18.877
COPD	0.153	0.556	0.783	1.165	0.392	3.467
rEF	0.133	0.033	0.00	1.142	1.07	1.219
LVH	−0.5	0.477	0.294	0.606	0.238	1.544
LVDD	−0.467	0.405	0.249	0.627	0.284	1.386
LA	0.002	0.008	0.799	1.002	0.987	1.017
HbA1c	−0.743	0.315	0.019	0.476	0.256	0.883
Glucose	−0.008	0.003	0.005	0.992	0.987	0.998
Creatinine	−0.346	0.256	0.177	0.708	0.428	1.169
Na	0.159	0.038	0.00	1.173	1.088	1.264
AST	−0.005	0.007	0.497	0.995	0.981	1.009
ALT	0.007	0.004	0.087	1.007	0.999	1.015
ALBUMİN	0.022	0.023	0.34	1.022	0.977	1.07
T-Cholesterol	−0.001	0.002	0.803	0.999	0.995	1.004
LDL-C	−0.002	0.003	0.493	0.998	0.992	1.004
HDL-C	0.008	0.008	0.281	1.008	0.993	1.024
VLDL-C	0	0	0.685	1	1	1
Triglyceride	0	0.001	0.867	1	0.999	1.002
CRP	0.014	0.009	0.132	1.014	0.996	1.033
PLT	0	0	0.763	1	1	1
cQT	−0.017	0.004	0.00	0.983	0.976	0.99
CRPtoAlb	0.349	0.479	0.466	1.418	0.553	3.631
PLR	0	0.003	0.903	1	0.993	1.006
TGI	−0.712	0.385	0.064	0.491	0.231	1.044

AF: Atrial fibrillation. HT: hypertension. DM: diabetes mellitus. CKD: chronic kidney disease. COPD: chronic obstructive pulmonary disease. rEF: reduced ejection fraction. LVH: left ventricular hypertrophy. LVDD: left ventricular diastolic dysfunction. LA: left atrium. HbA1c: hemoglobin A1C. Na: Natrium(sodium). K: potassium. AST: aspartate aminotransferase. ALT: alanine aminotransferase. T-Cholesterol: total cholesterol. LDL-C: LDL cholesterol. HDL-C: HDLcholesterol. VLDL-C: VLDL cholesterol. CRP: C-reactive protein. PLT: platelet. cQT: corrected QT. CRPtoAlb: C-reactive protein/albumin ratio. PLR: platelet lymphocyte ratio. TGI: triglyceride–glucose index.

**Table 3:** Multivariate logistic regression analysis of factors associated with coronary slow flow.

	B	S.E.	*p*	OR	95% C.I. for EXP(B)	
					Lower	Upper
Age	−0.021	0.021	0.310	0.979	0.939	1.020
HT	−0.827	0.500	0.098	0.437	0.164	1.165
DM	−0.160	0.667	0.810	0.852	0.230	3.151
rEF	0.137	0.078	0.082	1.147	0.983	1.339
HbA1c	−0.003	0.499	0.995	0.997	0.375	2.649
Glucose	−0.001	0.004	0.845	0.999	0.990	1.008
NA	−0.008	0.080	0.915	0.991	0.847	1.161
cQT	0.005	0.008	0.500	1.005	0.990	1.020
TGI	−0.697	0.466	0.134	0.498	0.200	1.241

HT: hypertension. DM: diabetes mellitus. rEF: reduced ejection fraction. HbA1c: hemoglobin A1C. Na: natrium(sodium). cQT: corrected QT. TGI: triglyceride–glucose index.

## Data Availability

The datasets generated and/or analyzed during the current study are available from the corresponding author on reasonable request.

## Abbreviations

The following abbreviations are used in this manuscript:

IR	Insulin Resistance
CSF	Slow Flow Phenomenon
TGI	Triglyceride–Glucose Index
NCF	Normal Coronary Flow
CRP	C-Reactive Protein
HbA1c	Glycated Hemoglobin
TIMI	Thrombolysis in Myocardial Infarction
cTFC	Corrected TIMI Frame Count
CVD	Cardiovascular Disease
MI	Myocardial Infarction
CABG	Coronary Artery Bypass Grafting
PCI	Percutaneous Coronary Intervention
LVH	Left Ventricular Hypertrophy
LVDD	Left Ventricular Diastolic Dysfunction
AF	Atrial Fibrillation
HT	Hypertension
DM	Diabetes Mellitus
COPD	Chronic Obstructive Pulmonary Disease
CKD	Chronic Kidney Disease
GCP	Good Clinical Practice
RCA	Right Coronary Artery
LAD	Left Anterior Descending Artery
LCx	Left Circumflex Artery
LAO	Left Anterior Oblique
CSFP	Coronary Slow Flow Phenomenon
LVEF	Left Ventricular Ejection Fraction
INOCA	Ischemia with Non-Obstructive Coronary Arteries
MACE	Major Adverse Cardiac Events
CAD	Coronary Artery Disease
ROC	Receiver Operating Characteristic
LDL-C	Low-Density Lipoprotein Cholesterol
ApoB	Apolipoprotein-B
ESC	European Society of Cardiology
